# Dual transcriptomic analysis reveals metabolic changes associated with differential persistence of human pathogenic bacteria in leaves of Arabidopsis and lettuce

**DOI:** 10.1093/g3journal/jkab331

**Published:** 2021-09-22

**Authors:** Cristián Jacob, André C Velásquez, Nikhil A Josh, Matthew Settles, Sheng Yang He, Maeli Melotto

**Affiliations:** 1 Department of Plant Sciences, University of California, Davis, Davis, CA 95616, USA; 2 Department of Plant Sciences, Horticulture and Agronomy Graduate Group, University of California, Davis, Davis, CA 95616, USA; 3 Departamento de Ciencias Vegetales, Facultad de Agronomía e Ingeniería Forestal, Pontificia Universidad Católica de Chile, Santiago 7820436, Chile; 4 Department of Biology, Howard Hughes Medical Institute, Duke University, Durham, NC 27708, USA; 5 Bioinformatics Core Facility in the Genome Center, University of California, Davis, Davis, CA 95616, USA; 6 Department of Biology, Duke University, Durham, NC 27708, USA

**Keywords:** dual transcriptomic profiling, *Salmonella enterica*, *Escherichia coli* O157:H7, disease outbreak, food safety

## Abstract

Understanding the molecular determinants underlying the interaction between the leaf and human pathogenic bacteria is key to provide the foundation to develop science-based strategies to prevent or decrease the pathogen contamination of leafy greens. In this study, we conducted a dual RNA-sequencing analysis to simultaneously define changes in the transcriptomic profiles of the plant and the bacterium when they come in contact. We used an economically relevant vegetable crop, lettuce (*Lactuca sativa* L. cultivar Salinas), and a model plant, *Arabidopsis thaliana* Col-0, as well as two pathogenic bacterial strains that cause disease outbreaks associated with fresh produce, *Escherichia coli* O157:H7 and *Salmonella enterica* serovar Typhimurium 14028s (STm 14028s). We observed commonalities and specificities in the modulation of biological processes between Arabidopsis and lettuce and between O157:H7 and STm 14028s during early stages of the interaction. We detected a larger alteration of gene expression at the whole transcriptome level in lettuce and Arabidopsis at 24 h post inoculation with STm 14028s compared to that with O157:H7. In addition, bacterial transcriptomic adjustments were substantially larger in Arabidopsis than in lettuce. Bacterial transcriptome was affected at a larger extent in the first 4 h compared to the subsequent 20 h after inoculation. Overall, we gained valuable knowledge about the responses and counter-responses of both bacterial pathogen and plant host when these bacteria are residing in the leaf intercellular space. These findings and the public genomic resources generated in this study are valuable for additional data mining.

## Introduction

Globally, according to the World Health Organization (WHO), the most frequent causes of foodborne illness are diarrheal disease agents, primarily non-typhoidal *Salmonella enterica* and enteropathogenic *Escherichia coli*, which in 2010 caused an estimated 230,000 deaths (WHO 2015). Outbreaks of *S. enterica* and pathogenic *E. coli* associated with the intake of uncooked produce are reported every year in Europe (Callejón *et al.* 2015) and in the United States (Bennett *et al.* 2018). For example, from 1998 to 2013, there were 972 raw produce-linked outbreaks reported to the Foodborne Disease Outbreak Surveillance System managed by the Center for Disease Control and Prevention, which accounted for 34,674 illnesses, 2,315 hospitalizations, and 72 deaths in the United States (Bennett *et al.* 2018). In particular, leafy greens products from California, mainly lettuce and spinach, were involved in 46 outbreaks from 1996 to 2016, where the most frequent microbiological hazards were *E. coli and Salmonella* (Turner *et al.* 2019).

Over the last two decades, an extraordinary effort has been made to gain an understanding of the biological processes underlying the contamination of fresh produce by *S. enterica and E. coli* (Hernández-Reyes and Schikora 2013; Sapers and Doyle 2014). Specifically, biochemical, molecular, and genetic studies have revealed a significant contribution of the plant innate immune response against human pathogenic bacteria in the phyllosphere (Schikora *et al.* 2011; Melotto *et al.* 2014; Jo and Park 2019). After exposure to *Salmonella* or *E. coli* serotype O157:H7 (hereafter O157:H7), pattern-triggered immunity responses (*i.e.*, reactive oxygen species burst, stomatal closure, activation of mitogen-activated protein kinases, ethylene, salicylic acid, and jasmonic acid signaling, expression of pathogenicity-related proteins, and callose deposition) have been observed in Arabidopsis (Thilmony *et al.* 2006; Schikora *et al.* 2008; Garcia *et al.* 2014), tobacco (Shirron and Yaron 2011), lettuce (Roy *et al.* 2013; Jacob and Melotto 2019; Jechalke *et al.* 2019), and *Medicago truncatula* (Jayaraman *et al.* 2014). Research has also elucidated numerous molecular determinants involved in the colonization of bacteria in/on leaves (Holden *et al.* 2009; Brandl *et al.* 2013; Yaron and Römling 2014). For instance, components of the bacterial extracellular matrix, such as curli, cellulose, and colanic acid (CA) have a significant role in the attachment and/or persistence of O157:H7 in lettuce (Fink *et al.* 2012) and spinach (Saldaña *et al.* 2011; Macarisin *et al.* 2013) and of *S. enterica* serovars Enteritidis and Newport in alfalfa sprouts (Barak *et al.* 2007). In addition, significant modulation of the expression of genes related to antimicrobial resistance, oxidative stress, motility, and carbon, nitrogen, and sulfur transport and metabolism has been observed in O157:H7 and *S. enterica* serovar Weltevreden after inoculation on lettuce leaves (Fink *et al.* 2012; van der Linden *et al.* 2016) and alfalfa sprouts (Brankatschk *et al.* 2014), respectively. Furthermore, the contribution of structural type 3 secretion system (T3SS) and type 3 effector (T3E) proteins in *Salmonella* adhesion, internalization, and persistence in/on the phyllosphere has been widely investigated (Schikora *et al.* 2011; Shirron and Yaron 2011; Chalupowicz *et al.* 2018; Montano *et al.* 2020). Nevertheless, the specific roles of certain T3SS and T3E proteins remain controversial and might be pathosystem specific.

Microarray- and RNA-seq-based transcriptomic studies have contributed substantially to unravelling the molecular processes occurring during the interaction between the plant and *S. enterica* or *E. coli* (Thilmony *et al.* 2006; Schikora *et al.* 2011; Fink *et al.* 2012; Brankatschk *et al.* 2014; Garcia *et al.* 2014; Jayaraman *et al.* 2014; Landstorfer *et al.* 2014; Van der Linden *et al.* 2016; Jechalke *et al.* 2019). Nonetheless, these studies have limited their genome-wide gene expression analyses to only one of the organisms involved in the interaction, the host or the pathogen, over time. With current high-throughput RNA-seq tools and bioinformatics resources, dual transcriptomic studies can be conducted, in which deep transcriptomic profiles of the microbial and the eukaryotic host cells are simultaneously captured (Saliba *et al.* 2017; Westermann *et al.* 2017). Bioinformatics pipelines have been developed and described in detail to analyze dual RNA-seq experiments where total reads are preprocessed, mapped to each or in parallel to both reference genomes, counted, and finally normalized for downstream analysis (Marsh *et al.* 2018; Nobori *et al.* 2018). Dual RNA-seq technical approaches have been applied in a variety of host/pathogen combinations, such as *Mus musculus*/*Streptococcus pneumoniae* (Ritchie and Evans 2019), *Salmo salar*/*Piscirickettsia salmonis* (Valenzuela-Miranda and Gallardo-Escárate 2018), *Eucalyptus nitens*/*Phytophthora cinnamomi* (Meyer *et al.* 2016), and *M. truncatula*/*Erysiphe pisi* (Gupta *et al.* 2020).

In our study, we aimed to simultaneously obtain the transcriptomic profiles of the two human pathogenic bacteria most frequently linked to disease outbreaks, *Salmonella and E. coli*, with the model plant Arabidopsis and the vegetable crop lettuce, which has been the vehicle for disease outbreaks. These multiple datasets enabled us to explore shared and unique metabolic processes that are modulated at different times in several bacterium–plant combinations. In addition, we identified multiple bacterial and plant genes with potential key roles in the leaf colonization by these two human pathogenic bacteria.

## Materials and methods

### Plant material and growth conditions

Arabidopsis (*Arabidopsis thaliana* [L.] Heynh. ecotype Col-0) and lettuce (*Lactuca sativa* L. cultivar Salinas) were grown as previously described (Jacob *et al.* 2017; Jacob and Melotto 2019). The iceberg lettuce cultivar Salinas was chosen for this study because it is extensively used in genetics studies and breeding efforts; its genome is also sequenced and annotated as a reference for this species (Reyes-Chin-Wo *et al.* 2017). Arabidopsis seeds were incubated for 2 days at 4°C for stratification. Lettuce seeds were germinated directly on water-soaked paper in square Petri dishes and maintained at 20°C for 2 days. Subsequently, Arabidopsis seeds were sown in 7.62 cm^2^ pots (3″; Kord Products, Toronto, Canada) containing a soil mix (Sun Gro^®^ Sunshine^®^ #1 Grower Mix with RESiLIENCE™, Agawam, MA, USA) and germinated lettuce seeds were transferred to the same pot type filled with the same soil mix. The soil used for Arabidopsis was previously soaked overnight with a fungus gnat prevention solution, 1 g/l Gnatrol WDG (Valent, Canada), and to avoid the contact of the leaves with the soil, a layer of fine vermiculite was added on the surface, which was then covered with a fiberglass mesh screen. Arabidopsis growth chamber conditions were 22 ± 1°C, 65 ± 5% relative humidity (RH), and photosynthetically active light intensity of 90 ± 10 μmol/m^2^/s. Lettuce plants were grown at 19 ± 1°C, 75 ± 4% RH, and photosynthetically active light intensity of 240 ± 10 μmol/m^2^/s. Both species were grown with a 12-h photoperiod. One liter of tap water was added to the tray two to three times per week depending on the developmental stage of the plants. At 10 days post germination, lettuce plants were fertilized with 0.05 g/plant of fertilizer (Multi-Purpose 19-11-21; Peters^®^Excel, OH, USA) dissolved in the irrigation water.

### Bacterial strains and inoculation procedure

The non-typhoid *S. enterica* subspecies *enterica* serovar Typhimurium strain 14028s (Porwollik *et al.* 2014) and the enterohemorrhagic *E. coli* serotype O157:H7 strain 86-24 (Sperandio *et al.* 2001) were grown in Low Salt Luria-Bertani medium (10 g/l tryptone, 5 g/l yeast extract, 5 g/l NaCl, pH 7.0) at 28°C. Medium supplemented with 50 µg/ml of streptomycin was used to grow O157:H7. To minimize bacterial stress due to centrifugation, the inoculum was prepared by directly transferring the bacterial cultures from the solid medium into a sterile 1 mM MgCl_2_ aqueous solution. The inoculum was infiltrated into the intercellular space of the leaf using a needleless syringe, as previously described by Katagiri *et al.* (2002). Inoculations were conducted in 3.5-week-old Arabidopsis plants and 4-week-old lettuce plants (Supplementary Figure S1, A and B). To determine the effect of inoculum concentration on bacterial persistence and optimum recovery of bacterium transcripts for downstream analyses, Arabidopsis plants were inoculated with four levels of bacterial inoculum, 1 × 10^6^, 10^7^, 10^8^, or 10^9^ CFU/ml. For RNA extraction, Arabidopsis plants were inoculated with a low (5 × 10^6^ CFU/ml) and a high (1 × 10^9^ CFU/ml) inoculum concentration, while lettuce plants were inoculated with a high inoculum concentration only. Mock inoculation with 1 mM MgCl_2_ was used as the negative control. Each plant was used to determine bacterial population size and extract RNA for transcriptomic profiling. The analysis workflow is depicted in Supplementary Figure S1C.

### Bacterial enumeration

Leaves from the same plants used for RNA extraction were collected to determine the size of the apoplastic bacterial population following the serial dilution method as previously described (Jacob *et al.* 2017).

### RNA extraction and quality assessment

Total RNA was extracted from fresh inoculum immediately before the inoculations. Three 1 ml samples (n = 3) were centrifuged at 3000 × *g* for 2 min at 20°C to recover bacterial cell pellets that were immediately used for RNA extraction using the RiboPure™-Bacteria Kit (Ambion, Austin TX, USA) according to the manufacturer’s instructions.

RNA from inoculated leaves was extracted at 4 and 24 h post inoculation (HPI). Whole leaves (Arabidopsis) or leaf pieces (lettuce) were excised from three separate plants for each sampling point for each treatment (n = 3). Immediately after collection, leaf material was placed into 2 ml self-standing, impact-resistant tubes (USA Scientific Inc., Ocala, FL, USA) containing three Zirconium beads (3 mm diameter; Glen Mills Inc., Clifton, NJ, USA), frozen in liquid nitrogen, and stored at −80°C. Leaf tissue was mechanically disrupted twice using a tissue grinder (Mixer Mill MM 400; Retsch, Haan, Germany) for 1 min at 30 Hz; the tissue was kept frozen throughout the procedure. Tubes were transferred to ice after tissue disruption and 1 ml of TRIzol reagent (Thermo Fisher Scientific, Waltham, MA, USA) was added. After incubation for 5 min, 200 µl of chloroform was added, the sample was vortexed for 20 seconds, and then centrifuged for 15 min at 16,000 × *g* and 4°C. The clear phase, normally approximately 500 µl, was transferred to a new tube and an equal volume of refrigerated RNase-free 100% (v/v) ethanol was added. The solution was then loaded into a clean-up column of the Direct-Zol™ RNA Miniprep kit (Zymo Research, Irvine, CA, USA) for RNA purification according to the manufacturer’s protocols.

All samples were subjected to a DNase treatment with the Turbo DNA-free kit (Ambion, Austin, TX, USA) and total RNA was quantified using a Qubit 3.0 fluorometer (Life Technologies, Carlsbad, CA, USA) and NanoDrop 2000c spectrophotometer (Thermo Scientific, Waltham, MA, USA). RNA quality was also assessed using a 2100 Bioanalyzer (Agilent, Santa Clara, CA, USA) and 1% agarose electrophoresis containing 1% (v/v) bleach (Clorox, Oakland, CA, USA) as recommended by Aranda *et al.* (2012).

### Library preparation and sequencing

Libraries from Arabidopsis samples were prepared using the Ovation Universal RNA-Seq System (NuGEN, San Carlos, CA, USA), with additional custom AnyDeplete (NuGEN, San Carlos, CA, USA) probes (Nobori *et al.* 2018). RNA extracted from leaves inoculated with 1 × 10^9^ CFU/ml were subjected to bacterial rRNA depletion with the Ribo-Zero™ rRNA Removal Kit, Bacteria (Illumina, San Diego, CA, USA). Libraries from lettuce samples were prepared using both Bacteria and Plant Ribo-Zero™ rRNA Removal Kits (Illumina, San Diego, CA, USA). Libraries from bacterial inoculum were prepared using the Ribo-Zero™ rRNA Removal Kits, Bacteria (Illumina, San Diego, CA, USA).

Sequencing libraries were quality controlled and quantified using a combination of Qubit dsDNA HS (Thermo Fisher Scientific, Waltham, MA, USA) and Advanced Analytical Fragment Analyzer High Sensitivity NGS DNA (Agilent, Santa Clara, CA, USA) assays. Sequencing was conducted in an Illumina HiSeq 4000 flow cell with a 1 × 50 -bp single read format using HiSeq 4000 SBS reagents. Base calling was done by Illumina Real Time Analysis (RTA) v2.7.7 and the RTA output was demultiplexed and converted to FastQ format with the Illumina Bcl2fastq v2.19.1 software.

### Reads processing

Preprocessing of reads, including filtering of rRNA, adapter trimming, and quality-based trimming (a Phred score of 20 was used as the threshold for the average score in a sliding window, with a minimum length of 20 bp) was conducted using the HTStream (https://github.com/s4hts/HTStream; accessed October, 2021) software. Subsequently, reads were simultaneously mapped to the sequenced genomes of three species, one plant species [Arabidopsis (TAIR10) or lettuce (LsV8)] and the two bacterial species [O157:H7 (GCA_000978845.1) and STm 14028s (GCA_000783815.1)] using the default parameter of the STAR RNA-Seq aligner version 2.5.2b. This approach was conducted to allow for the best alignment call for each read. Owing to the high redundancy of the bacterial genomes, the same read can map to both genomes if mapping were not done simultaneously, increasing the number of false positive alignments. Simultaneous mapping to all three genomes allows the aligner software to find the best match in only one genome providing the best alignment possible for this large dataset. The number of reads mapped to each genome is listed in Supplementary Table S1. Finally, the number of reads assigned to each gene was determined and read counts were normalized with the Log_2_ counts per million (CPM) normalization method (Law *et al.* 2018).

### Data analysis

To assess the interaction between the factors “inoculum concentration” and “days post inoculation” on the bacterial population, the data were subjected to a two-way analysis of variance (ANOVA) for each combination of plant and bacterium. Due to the significant interaction between these two factors (*P* < 0.0001) in all plant-bacterium combinations, we proceeded with assessing statistically significant changes in the bacterial population at the sampling points for each inoculum concentration within plant–bacterium combinations using ANOVA, followed by comparisons of multiple means using Tukey’s test with a significance threshold of α = 0.05.

The variation among replicates was evaluated with the correlation analyses multidimensional scaling (MDS) plot (Supplementary Figure S2) and Spearman’s correlation test (Supplementary Figure S3) using the normalized read counts of each sample. All MDS plots (Supplementary Figure S2) show data from all treatments due to the simultaneous mapping of reads to both plant and bacterial genomes described above. Plots were generated with the R packages edgeR version 3.18.1 (MDS) and heatmap.2 (Spearman’s correlation). Genes with expression values of 1 CPM or greater in at least five samples were kept for downstream analysis.

Differential expression was tested using a single factor ANOVA model in the limma package v3.32.10 of R v3.4.4. (Law *et al.* 2018). Significantly differentially expressed genes (DEGs) were considered when genes had a Benjamini–Hochberg false discovery rate (Benjamini and Hochberg 1995) with an adjusted *P*-value of <0.001 for plant genes and <0.05 for bacterial genes and with a Log_2_ fold change (FC) >1 or <−1. Unique and shared significantly up or downregulated genes between treatments were obtained using the software InteractiVenn (http://www.interactivenn.net/; accessed October, 2021; Heberle *et al.* 2015) and plots showing the number of intersecting DEGs were constructed based on the UpSet plots from Lex *et al.* (2014).

Gene ontology (GO) enrichment analysis of significant DEGs of Arabidopsis and lettuce was conducted using the GO Consortium tool PANTHER14.1 Classification System (Mi *et al*., 2021) and Blast2GO software (Götz *et al.* 2008), respectively. Additionally, we used the corresponding Arabidopsis orthologous genes of the lettuce genes reported by Reyes-Chin-Wo *et al.* (2017). Predicted lettuce proteins were BLASTed against *A. thaliana* TAIR10 proteins with BLASTp (threshold E-value = 1 × e^−10^) to find homologous sequences. Out of the total number of predicted lettuce genes, 29,681 (76.27%) genes showed similarity to Arabidopsis TAIR10 annotations (Reyes-Chin-Wo *et al.* 2017).

Metabolic pathway reconstruction and hierarchical clustering analyses of plant DEGs were performed with the MapMan 3.5.1R2 (Thimm *et al.* 2004) and heatmap.2 R package, respectively, using default settings. To analyze the relationship between treatments, comprehensive lists of 7112 Arabidopsis genes and 7469 lettuce genes containing all DEGs throughout the samples were used in the hierarchical cluster analysis. Using the Arabidopsis orthologs of lettuce genes we were able to compare all the treatments according to genes involved in biological processes related to primary and secondary metabolism and biotic stress, which were selected based on MapMan 3.5.1R2 classification and genes comprising enriched GO terms.

To analyze the relationship between bacterial transcriptomic changes among the treatments, we conducted hierarchical clustering analyses using lists comprising all DEGs throughout the O157:H7 samples (2420 DEGs) and the STm 14028s samples (2887 DEGs) using the R package heatmap.2. Metabolic reconstruction of bacterial responses to different nutritional environments were made using the KEGG Mapper tool (Kanehisa and Sato 2020; https://www.kegg.jp/kegg/mapper/reconstruct.html; accessed October, 2021). First, KEGG protein IDs were obtained using the protein sequences available in each bacterial genome annotation resources as an input for the KofamKOALA tool with the threshold E-value = 1 × e^−5^ (Aramaki *et al.* 2020; https://www.genome.jp/tools/kofamkoala/; accessed October, 2021). Second, the KEGG IDs were linked to the corresponding DEG. KEGG IDs were used as input for reconstructing pathways using KEGG Mapper. Finally, we used the mapping results to run a KEGG pathway enrichment analysis using the hypergeometric test for over-representation of success as previously described (Chen *et al.* 2015).

## Results and discussion

### Optimum inoculation dosage for dual transcriptomic analysis

**Figure 1:**
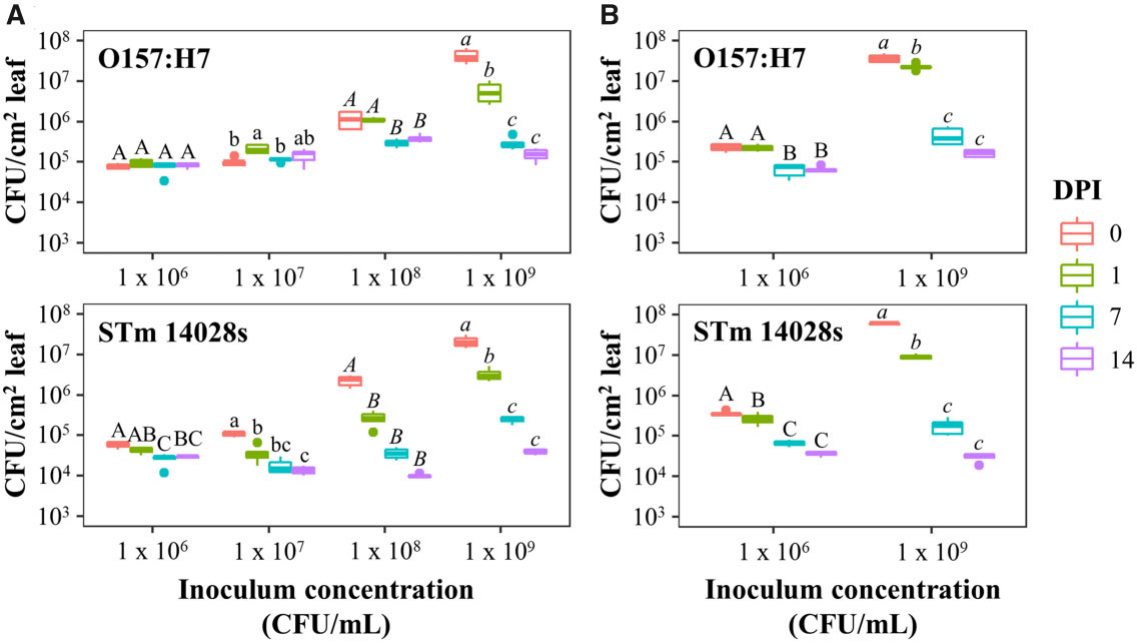
*Escherichia coli* O157:H7 and *Salmonella enterica* Typhimurium 14028s persistence in Arabidopsis (A) and lettuce (B) leaves after infiltration with different concentrations of inoculum (1 × 10^6^, 10^7^, 10^8^, or 10^9^ CFU/ml). Leaves were surface sterilized, except at 0 days post inoculation (DPI), and serial dilution plating was conducted to quantify the bacterial population in the intercellular space. Three plants were used for each sample point (n = 3). Different letters on the top of adjacent data points (*i.e.*, within the inoculum concentration groups) indicate significant statistical differences among the means, as calculated with ANOVA followed by Tukey’s test (α = 0.05).

To perform a dual transcriptomic analysis, it is crucial to estimate the relative concentration of both bacterial and host RNA in the sample. This estimate can inform the required sequencing depth and adjustments in the inoculation level to obtain enough bacterial read counts for downstream analyses (Westermann *et al.* 2017). Thus, we inoculated Arabidopsis and lettuce with a range of inoculum concentrations from 1 × 10^6^ to 10^9^ CFU/ml and assessed bacterial population kinetics over 14 days post-inoculation (DPI). Although the initial bacterial population sizes at the day of inoculation (0 DPI) of both O157:H7 and STm 14028s were different, at 14 DPI their population sizes reached comparable values independently of the inoculum concentration (Figure 1).

Previously, we observed that a bacterial population size of at least 1 × 10^6^ CFU/cm^2^ leaf is required to yield a sufficient number of bacterial reads for downstream bioinformatics analysis (Nobori *et al.* 2018). This level was only reached in leaves inoculated with 1 × 10^9^ CFU/ml at 0 and 1 DPI in both Arabidopsis and lettuce (Figure 1), resulting in a range of 5.1–11.9 and 1.9–4.6 million bacterial reads in leaf samples of Arabidopsis and lettuce, respectively (Supplementary Table S1), which is enough to detect significantly DEGs (Haas *et al.* 2012). Notably, inoculated leaves showed no macroscopic symptoms during the sampling period (Supplementary Figure S4). The use of a high inoculum concentration is not entirely unrealistic as focal contamination of leaves with fecal matter of super-shedder animals (*e.g.*, swine, cattle) or wild birds (*e.g.*, pheasants, gull, goose) can carry high levels of bacteria (1 × 10^7-9^ CFU/g feces; Brichta-Harhay *et al.* 2007; Gopinath *et al.* 2012; Smith *et al.* 2020), which may spread to adjacent plants in the field. Nonetheless, we also performed a transcriptomic analysis of Arabidopsis leaves inoculated with a substantially lower bacterial concentration (5 × 10^6^ CFU/ml) to assess the effect of inoculum concentrations in the plant transcriptomic profile.

### Transcriptomic changes in Arabidopsis after inoculation with low bacterial concentration

**Figure 2:**
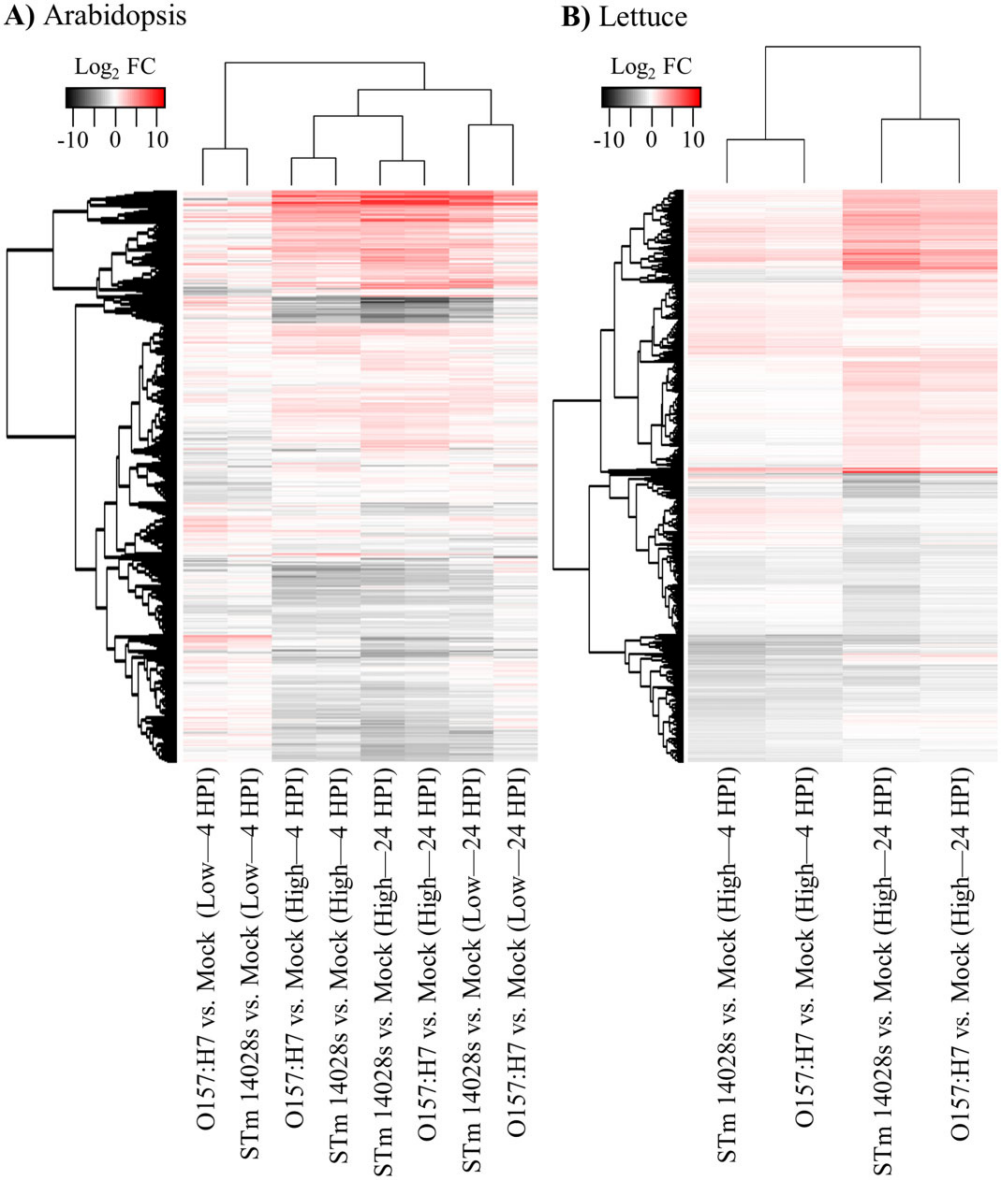
Hierarchical clustering analysis of differentially expressed genes (DEGs) in Arabidopsis (A) and lettuce (B) leaves at 4 and 24 h post inoculation (HPI) with low (5 × 10^6^ CFU/ml) or high (1 × 10^9^ CFU/ml) inoculum concentration of *Escherichia coli* O157:H7 and *Salmonella enterica* Typhimurium 14028s. Clustering was conducted using the heatmap.2 R package with default analysis settings. All DEGs were used as input for the hierarchical clustering analysis (7112 Arabidopsis genes and 7470 lettuce genes) and are listed in Dataset S03. FC, fold change.

The bacterial populations after infiltration with low concentration inoculum (5 × 10^6^ CFU/ml; Figure 1A) were insufficient to capture the number of bacterial reads for DEG analysis (Supplementary Table S1). Nevertheless, using this condition, we observed significant changes in the transcription of Arabidopsis genes at the whole-genome scale (Figure 2; Dataset S03). When comparing each bacterial treatment with the mock control at each time point, we observed that O157:H7 modulated a higher number (2343) of genes at 4 HPI and STm 14028s modulated a higher number of genes (3089) at 24 HPI (Table 1; see Dataset S01).

**TABLE 1.** Number of significantly differentially expressed genes (DEGs) in the plant (Arabidopsis and lettuce) and bacteria (Escherichia coli O157:H7 and Salmonella enterica ser. Typhimurium 14028s) at 4 and 24 hours post inoculation (HPI), as well as in the bacterium inoculum. Putative orthologs of lettuce DEGs were identified in Arabidopsis as reported by Reyes-Chin-Wo *et al*. 2017. Modulated genes identified with each comparison are listed in Dataset S01.

Inoculum concentration	Genome	Comparison	Induced genes	Repressed genes	Total DEGs
5 × 10^6^ CFU/ml	Arabidopsis	O157:H7 *vs* mock (4 HPI)	880	1443	2323
		O157:H7 *vs* mock (24 HPI)	523	482	1005
		STm 14028s *vs* mock (4 HPI)	136	623	759
		STm 14028s *vs* mock (24 HPI)	1470	1619	3089
1 × 10^9^ CFU/ml	Arabidopsis	O157:H7 *vs* mock (4 HPI)	2176	2200	4376
		O157:H7 *vs* mock (24 HPI)	2523	2294	4817
		STm 14028s *vs* mock (4 HPI)	2327	1918	4245
		STm 14028s *vs* mock (24 HPI)	2928	2810	5738
	O157:H7	4 HPI *vs* inoculum	781	1379	2160
		24 HPI *vs* 4 HPI	60	60	120
	STm 14028s	4 HPI *vs* inoculum	526	2041	2567
		24 HPI *vs* 4 HPI	175	59	234
1 × 10^9^ CFU/ml	Lettuce	O157:H7 *vs* mock (4 HPI)	691	999	1690
		O157:H7 *vs* mock (24 HPI)	2119	566	2685
		STm 14028s *vs* mock (4 HPI)	1502	1859	3361
		STm 14028s *vs* mock (24 HPI)	3124	2240	5364
	Arabidopsis (orthologs of lettuce DEGs)	O157:H7 *vs* mock (4 HPI)	446	730	1176
		O157:H7 *vs* mock (24 HPI)	1206	411	1617
		STm 14028s *vs* mock (4 HPI)	1042	1377	2419
		STm 14028s *vs* mock (24 HPI)	1928	1770	3698
	O157:H7	4 HPI *vs* inoculum	372	234	606
		24 HPI *vs* 4 HPI	63	101	164
	STm 14028s	4 HPI *vs* inoculum	249	526	775
		24 HPI *vs* 4 HPI	143	92	235

**Figure 3:**
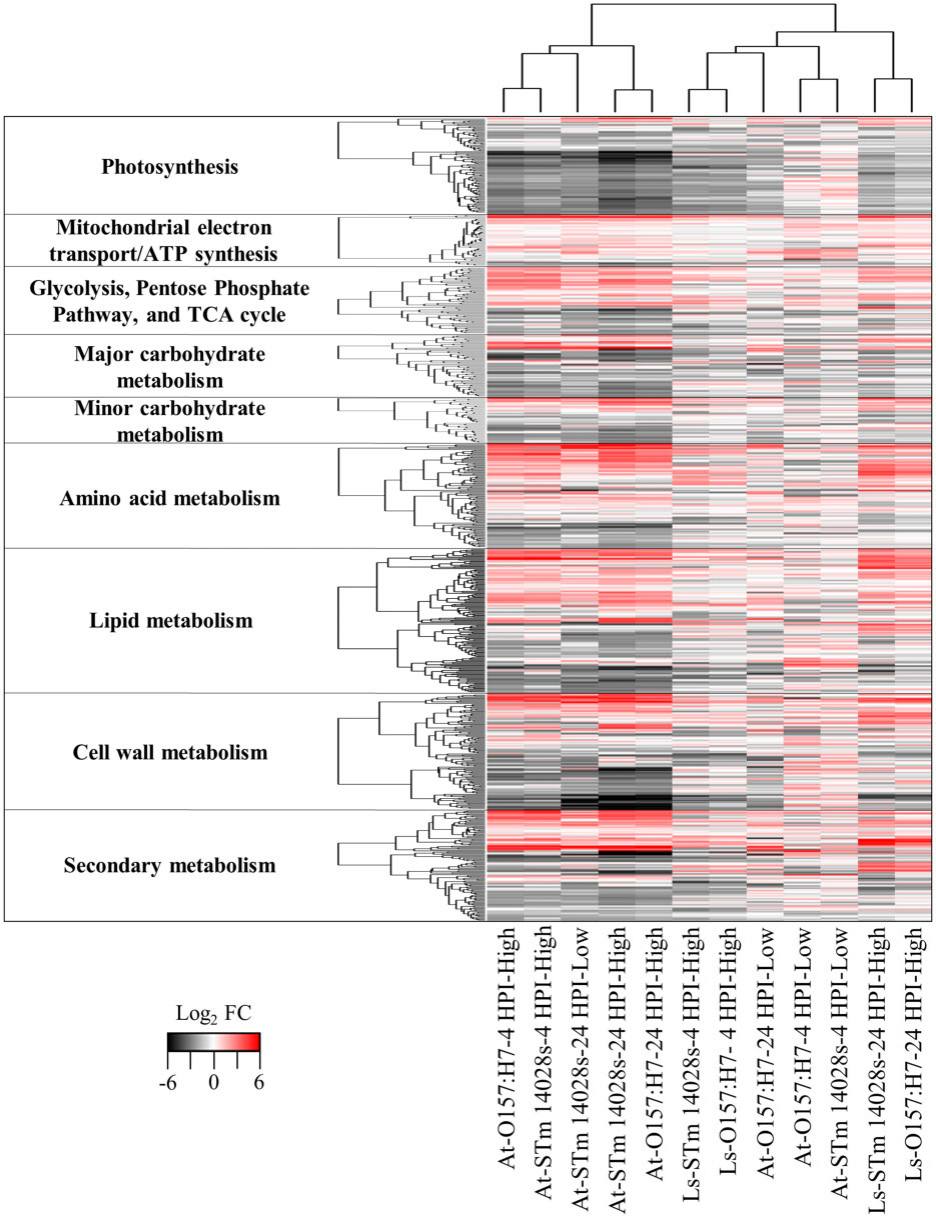
Hierarchical clustering analysis of differentially expressed genes (DEGs) involved in primary and secondary metabolism of Arabidopsis (At) and lettuce (Ls) at 4 and 24 h post inoculation (HPI) with a low (5 × 10^6^ CFU/ml) or high (1 × 10^9^ CFU/ml) inoculum concentration of *Escherichia coli* O157:H7 and *Salmonella enterica* Typhimurium 14028s. Clustering was conducted using the heatmap.2 R package with default analysis settings. The list of DEGs is described in Dataset S04. FC = fold change.

**Figure 4:**
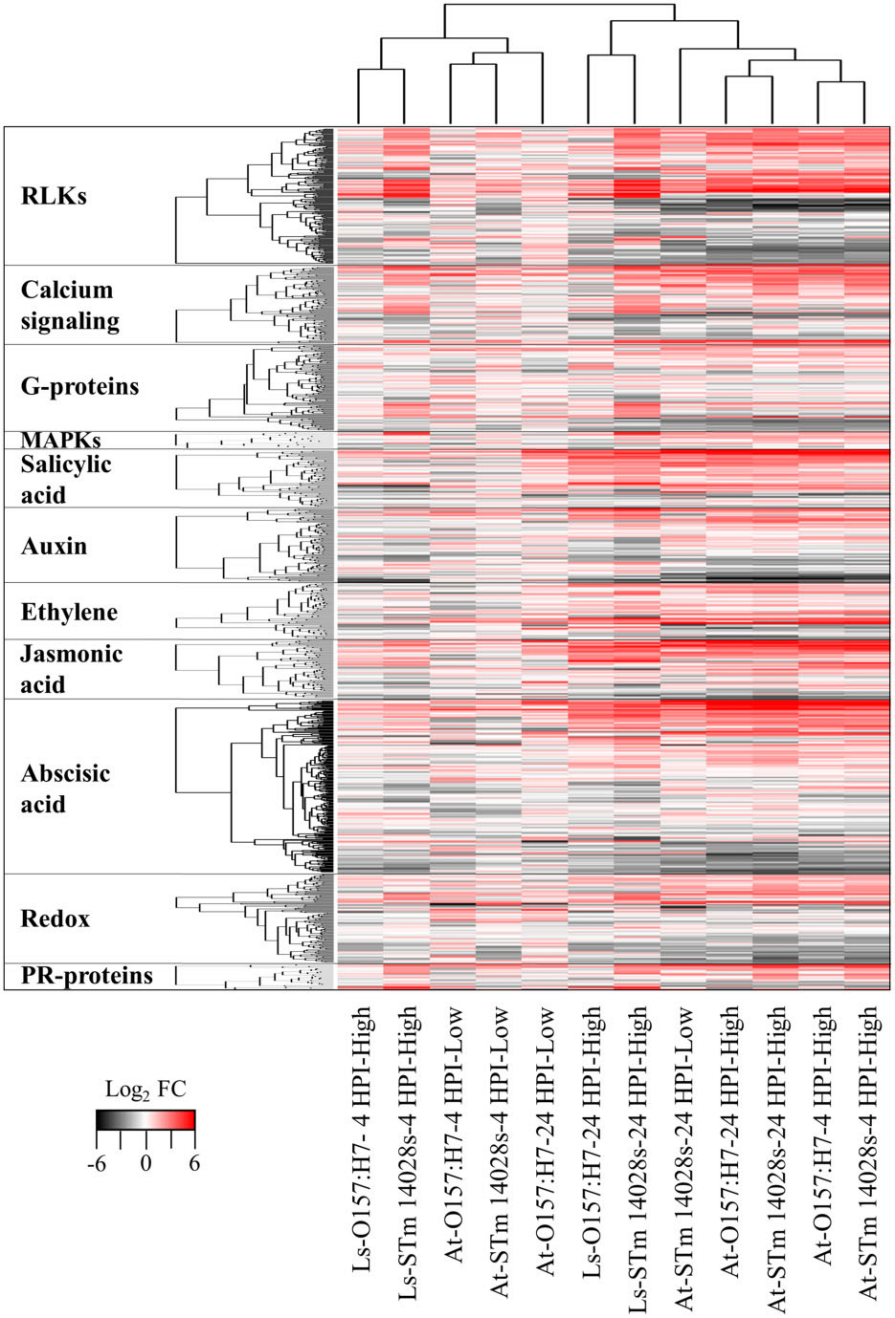
Hierarchical clustering analysis of differentially expressed genes (DEGs) involved in biotic stress responses of Arabidopsis (At) and lettuce (Ls) at 4 and 24 h post inoculation (HPI) with a low (5 × 10^6^ CFU/ml) or high (1 × 10^9^ CFU/ml) inoculum concentration of *Escherichia coli* O157:H7 and *Salmonella enterica* Typhimurium 14028s. Clustering was conducted using the heatmap.2 R package with default analysis settings. The list of DEG is described in Dataset S05. RLKs, receptor-like kinases; MAPKs, mitogen-activated protein kinases; PR, pathogenicity related; FC, fold change.

Broadly, the GO enrichment analysis revealed that these bacteria induce changes in Arabidopsis metabolism and stress responses when compared to the mock control (Dataset S02). These results prompted us to re-construct the metabolic maps for these processes and to compare the treatments through heatmaps and hierarchical analysis based on DEGs associated with metabolism and stress responses. These analyses showed that O157:H7 stimulated changes in a large amount of DEGs involved in primary metabolism (Figure 3 and Supplementary Figure S5; Dataset S04) and plant responses to biotic stress (Figure 4 and Supplementary Figure S6; Dataset S05) at 4 HPI, while STm 14028s modulated a higher number of genes in these categories at 24 HPI (Figures 3 and 4).

Plants inoculated with STm 14028s or O157:H7 exhibited an overall reduction of photosynthesis reactions, which was accompanied by a downregulation in the expression of genes associated with other primary metabolic pathways (Figure 3 and Supplementary Figure S5). Furthermore, Arabidopsis interaction with STm 14028s and O157:H7 significantly induced the expression of genes involved in aging and leaf senescence, suggesting a disruption of normal leaf growth and development. Plants inoculated with O157:H7 exhibited an induction of the genes *PYRUVATE, ORTHOPHOSPHATE DIKINASE* (PPDK), which is involved in nitrogen remobilization during leaf senescence (Taylor *et al.* 2010), and *SENESCENCE-ASSOCIATED PROTEIN* (*AAF*). The inoculation of leaves with STm 14028s caused the upregulation of the *SENESCENCE-ASSOCIATED GENES (SAG) 13*, *20*, *21*, and *101*.

It is widely theorized that during plant–pathogen interactions, growth–defense tradeoffs exist due to limited resources and that the balance between these two factors is regulated by phytohormone crosstalk (Huot *et al.* 2014). Accordingly, our analyses show that, at 24 HPI, most of the DEGs associated with plant defense signaling pathways are upregulated (Figure 4 and Supplementary Figure S6), which is also illustrated by the enriched GO terms associated with plant defenses among upregulated genes (Dataset S02).

#### Overlap analysis of DEG datasets

Next, we sought to determine the extent of the overlap between the DEGs identified by two-way comparisons (*i.e*., bacterium versus mock). This analysis revealed common and unique modulation of the Arabidopsis transcriptome by bacterial species (Supplementary Figure S7; Dataset S06).

##### Genes involved in Arabidopsis response to both STm 14028s and O157:H7

To identify Arabidopsis genes that were modulated by both bacteria, we conducted an intersect analysis summarized in Supplementary Figure S7A. At 4 HPI, 102 and 477 Arabidopsis genes were up- and downregulated respectively, whereas at 24 HPI, 243 and 304 were up- and downregulated, respectively (Dataset S06). Arabidopsis genes commonly upregulated by both STm 14028s and O157:H7 were associated with GO terms involving response against biotic and abiotic stressors as early as 4 HPI, which continued to be enriched until 24 HPI. At this later time point, additional GO terms associated with plant immune response and signaling (such as protein phosphorylation and folding) were enriched (Dataset S07). The genes commonly repressed by these pathogens belong to enriched GO terms related to gene expression, mRNA processing, and primary metabolism at 4 HPI, whereas photosynthesis, carbohydrate metabolism, and cold acclimation associated GO terms were enriched at 24 HPI (Dataset S07).

Common transcriptional modulation in plants exposed to various bacteria has been previously reported. For instance, approximately 30% of *M. truncatula* DEGs are commonly regulated in response to cocktails of O157:H7 strains and *S. enterica* serovars, with a large proportion of genes related to pathogen defense being regulated similarly by these bacteria (Jayaraman *et al.* 2014). Likewise, Schikora *et al.* (2011) detected 164 overlapping DEGs in 14-day-old Arabidopsis Col-0 seedlings at 2 HPI with STm 14028s, the non-pathogenic *E. coli* strain DH5α, and the phytopathogen *Pseudomonas syringae* pv. *tomato* DC3000. Our dataset has some overlap with the data reported by Schikora *et al.* (2011), in which 11.6 and 4.3% of the 164 DEGs observed by Schikora and collaborators were also differentially expressed in our plants at 4 HPI with O157:H7 and STm 14028s, respectively, while at 24 HPI, these values increased to 12.8 and 43.9% (Dataset S08). Altogether, these findings confirm the existence of a basal response to bacterial-caused stress in Arabidopsis Col-0 ecotype. However, certain biological processes were uniquely altered by either O157:H7 or STm 14028s as described below.

##### Genes involved in Arabidopsis response unique to either STm 14028s or O157:H7

In general, O157:H7 modulated more genes than STm 14028s at 4 HPI and the opposite was observed at 24 HPI (Table 1). Likewise, the number of genes uniquely modulated by O157:H7 was considerably larger than that modulated by STm 14028s at 4 HPI, whereas this trend was reversed at 24 HPI when the number of genes uniquely by STm 14028s was considerably larger (Dataset S06).

O157:H7 modulated process. The greatest metabolic changes in response to O157:H7 occurred at 4 HPI. Upregulated genes belong to cellular processes, macromolecular metabolism, and transport, whereas downregulated genes seem to be associated mostly with regulatory processes (Dataset S07). Various studies have shown that the induction of the plant cell secretory pathway is relevant for the trafficking of molecules involved in the interaction between plants and microbes (Wang and Dong 2011). At 24 HPI, O157:H7 downregulated genes in functional categories associated with response to abiotic stimulus and circadian rhythm. Only two GO terms were identified as significantly represented in the upregulated gene dataset (Dataset S07).

STm 14028 s modulated process. Interestingly, the only GO terms found to be enriched in the STm 14028s-4 HPI dataset are associated with RNA processing and gene expression, and these represented downregulated genes only. At 24 HPI, STm 14028s upregulated genes associated with cell wall modifications, hormone responses, and defense responses. The overall higher induction of genes related with defense response in Arabidopsis after inoculation with STm 14028s than O157:H7 (Figure 4 and Supplementary Figure S6) is correlated with the significant decline of STm 14028s population in the apoplast over time as compared to that of O157:H7 (Figure 1A). In addition, at this later time point, genes downregulated by STm 14028s are associated with response to light and photosynthesis (Dataset S07), possibly because of heighten defense response.

### Effects of increased bacterial population on the Arabidopsis transcriptome

Transcriptomic studies of plant responses during exposure to human pathogenic bacteria have been conducted under various experimental procedures and conditions. Although it is possible to use a very low inoculum concentration, such as 200 CFU/plant to inoculate *M. truncatula* seedlings (Jayaraman *et al.* 2014), a much higher inoculation dose is required for whole tissue transcriptomic analysis so that all plant cells are exposed to a uniform number of bacterial cells (Katagiri *et al.* 2002). Previous microarray-based studies were performed with 14-day-old Col-0 seedlings submerged in liquid medium containing STm 14028s or its *prgH−* mutant at a final concentration of 1.7 × 10^8^ and 2 × 10^8^ CFU/ml, respectively (Schikora *et al.* 2011; Garcia *et al.* 2014). Thilmony *et al.* (2006) vacuum-infiltrated 4-week-old Col-0 plants with 1 × 10^8^ CFU/ml of O157:H7 or its *fliC* mutant. However, these conditions would still not capture the bacterial transcriptomic for simultaneous analyses even with the latest Illumina technology. Thus, we sought to determine to what extent the inoculation dosage (5 × 10^6^ or 1 × 10^9^ CFU/ml) affects the plant transcriptome and aimed to analyze the bacterial metabolic changes *in planta*.

Arabidopsis leaves exposed to a high bacterial concentration exhibited an increase in both the number of total DEGs (Table 1) and the overall intensity of transcriptional regulation (Figure 2A). High-level inoculation also resulted in a similar response of Arabidopsis to both O157:H7 and STm 14028s when compared to plants inoculated with the low inoculum concentration (Figure 2A). The effect of different inoculum concentrations in the overall transcriptomic changes of Arabidopsis was less prominent at 24 HPI compared with that at 4 HPI (Figure 2A). The hierarchical analysis clustered the 24 HPI-low inoculum treatments in a subgroup closer to all high inoculum treatments than to the 4 HPI-low inoculum treatments (Figure 2A), suggesting that high inoculum induced faster and stronger response as expected. This is particularly evident in Arabidopsis leaves inoculated with low STm 14028s concentrations at 24 HPI that exhibited a pattern of gene transcriptional modulation very similar to those of samples from high inoculum concentration treatments (Figure 2A).

We next determined the extent of overlap between Arabidopsis DEGs identified in leaves exposed to the two inoculum concentrations. Overall, the proportion of DEGs shared between the two inoculation levels is low at 4 HPI for both bacteria, where approximately 10% of the DEGs in the low inoculum dataset is also present in the high inoculum dataset. However, at 24 HPI, more than 30 and 55% of DEGs in the low inoculum dataset is also present in the high inoculum dataset obtained from O157:H7 and STm 14028s, respectively (Supplementary Figure S7, C and D; Dataset S06).

To understand the biological function of genes commonly or uniquely differentially expressed between low and high inoculum concentration treatments, we conducted a GO enrichment analysis of each overlapping dataset (Dataset S09). This analysis showed that among the different datasets, most of the GO terms enriched among downregulated genes were related to photosynthesis and primary metabolism, while those enriched among upregulated genes were generally associated with stress response. The biological processes affected by different bacterial inoculum concentrations were similar; however, there was a relevant increment in the number of significantly DEGs belonging to primary and secondary metabolism (Figure 3; Dataset S04), as well as to biotic stress (Figure 4; Dataset S05) in Arabidopsis inoculated with the high inoculum concentration compared to those inoculated with the low inoculum concentration.

Taken together, these findings suggest that, with the purpose of identifying plant metabolic processes affected by O157:H7 and STm 14028s, high inoculation dosages are appropriate and provide the advantage of studying simultaneous changes in the bacterial transcriptomic profiles.

### Overlap between transcriptional profiles of Arabidopsis and lettuce exposed to O157:H7 and STm 14028s

Due to the relevance of lettuce for the market of fresh vegetables (Lucier and Parr 2020) and its frequent association with food safety incidents (Turner *et al.* 2019; Marshall *et al.* 2020), we analyzed changes in the transcriptomic profiles of leaves of the lettuce cultivar Salinas (Reyes-Chin-Wo *et al.* 2017) after exposure to either STm 14028s or O157:H7. With the exception of 24 HPI with STm 14028s, fewer lettuce genes were modulated by these bacteria as compared to those of Arabidopsis (Table 1). Furthermore, hierarchical clustering analysis of DEGs showed that, although datasets formed two clusters by time point, STm 14028s seems to induce stronger changes than O157:H7 indicated by the darker color at both time points (Figure 2B; Dataset S03).

The initial analyses described above were conducted with the original lettuce genes to call significant modulation (Dataset S01) and enriched GO terms using the Blast2GO software (Dataset S02). However, translating the lettuce genes into their corresponding Arabidopsis orthologs (Dataset S01) followed by functional annotation with the PANTHER14.1 tool proved to be more informative (Dataset S02). On average, 3.5 and 4.6 times more enriched GO terms were detected with PANTHER14.1 than Blast2GO at 4 and 24 HPI, respectively (Dataset S02), possibly due to the lower level of functional annotation of the lettuce genome as compared to the Arabidopsis genome (Reyes-Chin-Wo *et al.* 2017). It is important to note that an Arabidopsis ortholog could not be assigned to 23% of all lettuce genes (Reyes-Chin-Wo *et al.* 2017) and they were not included in further analyses. However, the conversion of lettuce genes to orthologous Arabidopsis genes provided two advantages, the increased functional annotation and the comparison of the bacterial-induced transcriptomic response between the two plant species as described below.

The number of lettuce genes modulated by STm 14028s was considerably larger than the number of O157:H7-modulated genes in comparison with the mock control (Table 1). This is in stark contrast with Arabidopsis, where both bacteria modulated a similar number of genes and the number of genes modulated was greater than that in lettuce (Table 1), suggesting distinct plant–bacterium interactions. These differences might be influenced by the total number of genes in the genome of each plant species: 27,655 in Arabidopsis (Cheng *et al.* 2017) and 38,919 in lettuce (Reyes-Chin-Wo *et al.* 2017). In addition, evolutionary forces (*i*.*e*., plant domestication and bacterial adaptation to different environments) may also play a role in the differential interaction between these organisms.

By identifying the putative Arabidopsis orthologs of lettuce DEGs (Dataset S01), it was possible to use them as input to reconstruct the major metabolic pathways and biotic stress response maps. This analysis again showed that STm 14028s modulates more genes than O157:H7 in both time points and most genes are downregulated at 4 HPI, whereas most genes are upregulated at 24 HPI (Supplementary Figures S8 and S9).

After identifying the DEGs in each plant species (bacterium versus mock control) at the two time points (Dataset S01), we conducted a pairwise comparison between different datasets. Overall, a larger overlap between the two plant species was observed in STm 14028s-modulated genes than O157:H7-modulated genes (Supplementary Figure S7, E and F; Dataset S06). Furthermore, the number of DEGs shared between the two time points was two or more times larger in Arabidopsis than lettuce (Supplementary Figure S7, E and F), suggesting that transcriptional changes in Arabidopsis last longer than in lettuce. Next, we analyzed the functional categories of DEGs that are shared by or unique to each plant species (Dataset S10).

#### Functional categories shared by both Arabidopsis and lettuce in response to human pathogens

Genes that were downregulated at 4 HPI in both plant species enriched GO terms involving photosynthesis (Figure 3; Dataset S04) and circadian rhythm after inoculation with O157:H7 or STm 14028s. The repression of photosynthesis contributes to the growth–defense tradeoffs during plant–pathogen interactions (Huot *et al.* 2014). Interestingly, both biological processes, photosynthesis and circadian rhythm, were largely repressed in lettuce cultivar Salinas inoculated with *B. cinerea* (De Cremer *et al.* 2013). The circadian clock in plants participates in the regulation of growth and development (*i.e.*, photosynthesis, stomatal movement, flowering, and senescence) and responses against abiotic and biotic stressors (Srivastava *et al.* 2019).

At 24 HPI, repressed genes shared by both plant species were associated with different biological processes depending on the human pathogenic bacteria. In the case of leaves inoculated with STm 14028s, all repressed genes at 24 HPI in both plant species were associated with photosynthesis (Figure 3; Dataset S04). In contrast, genes commonly downregulated in Arabidopsis and lettuce at 24 HPI with O157:H7 were related with growth and development (*i*.*e*., pattern specification process, response to auxin, and shoot system development) (Dataset S10).

Genes commonly upregulated in both plant species at 4 and 24 HPI with either STm 14028s or O157:H7 belong to the overrepresented GO categories related to plant defense responses (Figure 4; Dataset S05). However, the overall upregulation of genes associated with biotic stress in Arabidopsis leaves was higher compared to that observed in lettuce (Figure 4; Dataset S05). In addition, at 4 HPI, other biological processes, such as those represented by the GO terms *de novo* protein folding, endoplasmic reticulum unfolded protein response, and response to heat, were overrepresented by DEGs present in both plant species after inoculation with O157:H7 and STm 14028s.

#### Functional categories unique to lettuce in response to human pathogens

A biological process only induced in lettuce at 4 HPI is associated with ribosome biology and translation, such as the GO terms maturation of LSU- and SSU-rRNA from tricistronic rRNA transcript, ribosomal large subunit assembly, and translational elongation. Additionally, the GO term “phenylpropanoid biosynthetic process,” which is associated with chemical defenses against pathogens (Dixon *et al.* 2002), was only enriched among induced DEGs in lettuce, but not in Arabidopsis, after inoculation with STm 14028s or O157:H7 (Dataset S10). Similarly, one of the most relevant transcriptional changes caused by the inoculation with the necrotrophic pathogen *Botrytis cinerea* in 5-week-old lettuce cv. Salinas plants grown in soil was the induction of the phenylpropanoid pathway (De Cremer *et al.* 2013). Regulation of this pathway may be associated with lettuce response to a wide range of microbes.

The GO term “ethylene-activated signaling pathway” was specifically overrepresented among upregulated lettuce genes at 24 HPI with STm 14028s (Dataset S10). Various genes involved in the ethylene signaling pathway were also detected in the lettuce cv. Tizian after inoculation with STm 14028s (Jechalke *et al.* 2019), suggesting that ethylene response in lettuce may be specific to STm isolates.

Interestingly, the GO term “regulation of stomatal movement” was enriched only among repressed genes in lettuce at 4 HPI with STm 14028s (Dataset S10). Previously, we have shown that the stomatal closure in lettuce is sustained for several hours after inoculation with O157:H7; however, at 4 HPI leaves inoculated with STm strains SL1344 and 14028s exhibit a reopening of the stomatal pore (Roy *et al.* 2013; Montano *et al.* 2020). Although these STm strains regulate stomatal movement in Arabidopsis (Roy *et al.* 2013), this functional category was not enriched in Arabidopsis DEG datasets, suggesting that this response may be more prominent in lettuce than in Arabidopsis.

Overall, we observed significant differences between plant species in the modulation of genes within the broad GO term “biological functions,” in addition to a few specific GO terms exclusively enriched in Arabidopsis or lettuce samples after inoculation with the human pathogenic bacteria O157:H7 and STm 14028s.

### Bacterial responses to the Arabidopsis and lettuce apoplast environments

To assess potential metabolic changes that occur in the bacterial cells when they transitioned from the inoculum environment to the leaf apoplast, we compared the bacterial transcriptomic profiles in the inoculum with that at 4 HPI into the leaf apoplast (4 HPI *vs* inoculum; Table 1). In addition, subsequent changes in bacterial gene expression once in the apoplast were also assessed (24 HPI *vs* 4 HPI; Table 1).

Overall, both O157:H7 and STm 14028s exhibited a substantially larger transcription modulation at 4 HPI in the leaf environment compared to 24 HPI, both in number of DEGs (Table 1) and the extent of FC (Supplementary Figure S10; Dataset S11) in both plant species. This suggests that the metabolic changes taking place during the first hours in the leaf are crucial for the adaptation of the bacteria to the new environment and that most of these changes are sustained and only small readjustments are made in the subsequent 20 h. These observations were expected considering the change from a nutritional rich media to a relatively nutritional limited environment, the leaf apoplast. Furthermore, bacteria exposed to the apoplastic environment of Arabidopsis exhibited larger changes in the transcription of genes as compared to bacteria infiltrated into lettuce leaves, especially at 4 HPI (Supplementary Figure S10), suggesting that bacterial interactions with Arabidopsis are initially more intense than that with lettuce. These differences correlate with the overall stronger transcriptomic adjustments induced by O157:H7 and STm 14028s in Arabidopsis than in lettuce (Table 1; Figure 2; Dataset S03) and with the overall larger population of O157:H7 and STm 14028s during the first 24 HPI in lettuce compared with Arabidopsis (Figure 1).

#### Overlap analysis of bacterial DEG datasets in Arabidopsis and lettuce

**Figure 5:**
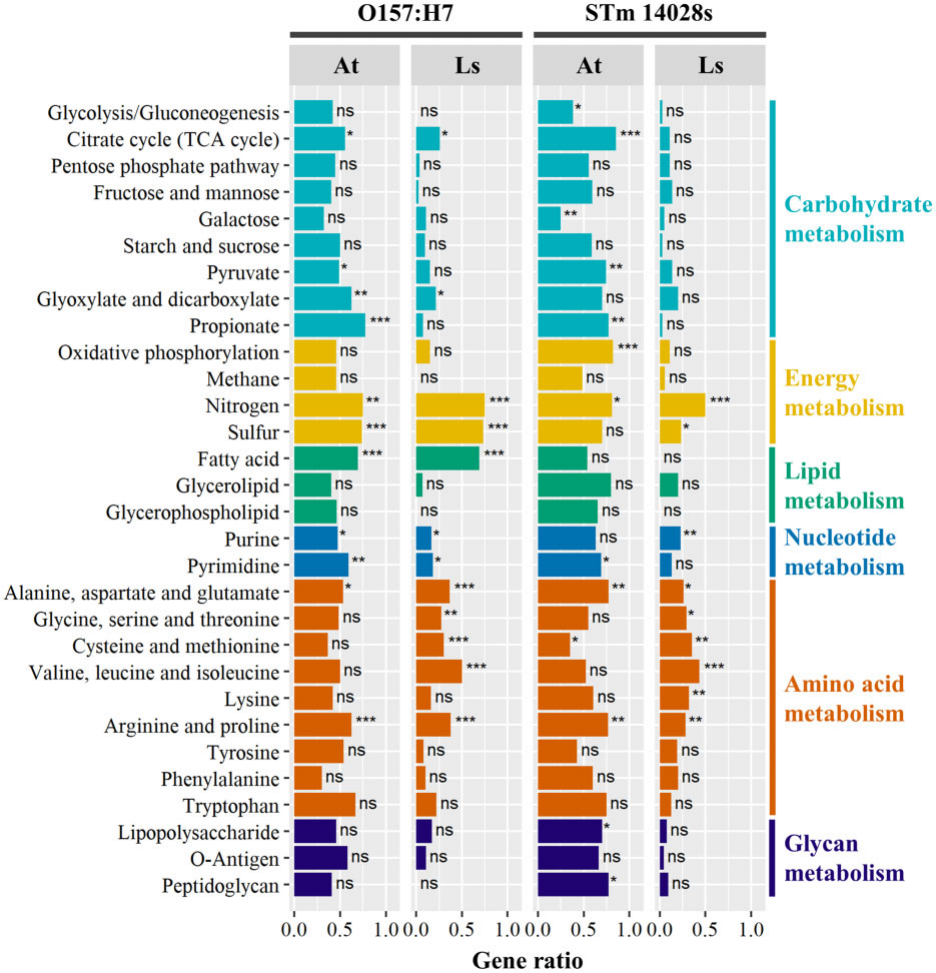
KEGG pathway enrichment analysis of genes differentially expressed by *Escherichia coli* O157:H7 and *Salmonella enterica* ser. Typhimurium 14028s after 4 h of inoculation in *Arabidopsis thaliana* (At) and lettuce (Ls = *Lactuca sativa* cv. Salinas). Gene ratio corresponds to the number of differentially expressed genes mapped to a KEGG pathway divided by the total number of genes comprising that KEGG pathway. To map the genes to the metabolic pathways, the protein sequences of the corresponding annotated genome were used to obtain the KEGG protein IDs using the KofamKOALA BLAST tool (https://www.genome.jp/tools/kofamkoala/). Protein sequences with an E-value lower than 10E^−5^ (listed in Dataset S12) were used as input for the KEGG Mapper Reconstruct Pathway tool (https://www.kegg.jp/kegg/tool/map_pathway.html). Enrichment analysis was conducted with the hypergeometric test (ns = *P*-value ≥ 0.5; *P-value < 0.5; **P-value < 0.01; ***P-value < 0.001).

Cellular metabolism in *Salmonella and E. coli* is complex and adaptable to different nutritional environments (AbuOun *et al.* 2009). Accordingly, we observed some commonalities and uniqueness in the transcriptome modification of both O157:H7 and STm 14028s when comparing the lettuce and Arabidopsis environments (Supplementary Figure S7, G and H; Dataset S06). To analyze the biological functionality of specific and shared transcriptomic changes of STm 14028s and O157:H7, we conducted a KEGG enrichment analysis through the hypergeometric test (Figure 5). This analysis revealed that, at 4 HPI, main metabolic pathways were significantly enriched depending upon plant-bacterium combinations (Figure 5).

Furthermore, as genes associated with specific metabolic processes are organized in operons and gene clusters in the bacterial genome, we assessed whether multiple genes in these regions were co-regulated (Supplementary Figure S10; Dataset S11) and created metabolic maps with predicted proteins encoded by the DEGs (Supplementary Figure S11; Dataset S12).

**Figure 6:**
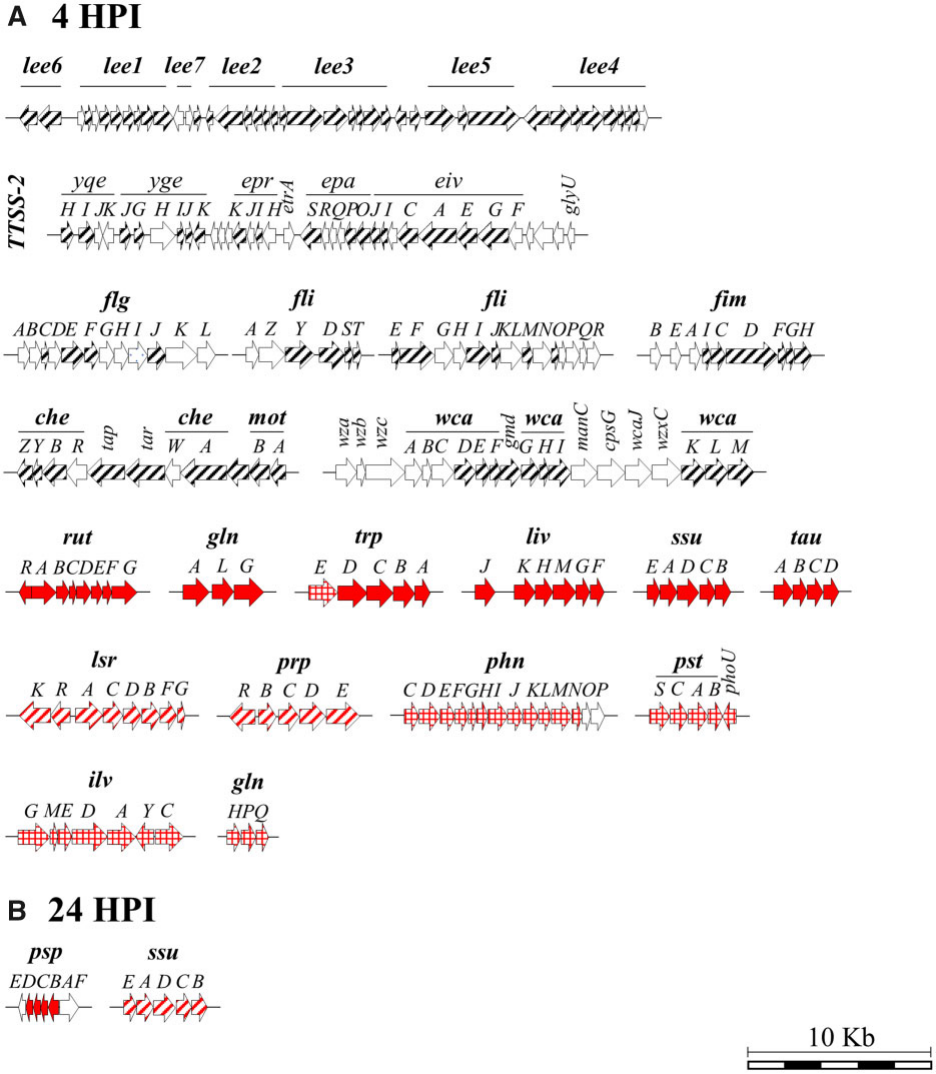
Major operons and gene clusters significantly modulated in *Escherichia coli* O157:H7 at 4 h (A) and 24 h (B) post inoculation (HPI). Locus of enterocyte effacement (*lee1-7*), type three secretion system 2 (*TTSS-2*), flagella (*flg and fli*), fimbria (*fim*), chemotaxis and motility (*che*-*mot*), colanic acid (*wca*), Rut pathway (*rut*), glutamine synthase (*glnALG*), tryptophan (*trp*), leucine, isoleucine, and valine transport system (*liv*), sulfonate-sulfur utilization (*ssu*), taurine utilization (*tau*), luxS-mediated quorum sensing (*lsr*), propionate (*prp*), organophosphonate utilization (*phn*), phosphate transporter (*pst*), isoleucine and valine biosynthetic pathway (*ilv*), glutamine transporter (*glnHPQ*), and phage shock proteins (*psp*). Genes differentially expressed in lettuce only (squared), Arabidopsis only (striped), and both lettuce and Arabidopsis (filled) are depicted within each operon and gene cluster. Genes colored in red, black, or white indicate upregulation, downregulation, or not differentially expressed, respectively. Schematic representation of operons and gene clusters was created based on the Ensembl Bacteria (https://bacteria.ensembl.org/index.html) genome browser. Description of the operons can be found in Dataset S11.

**Figure 7:**
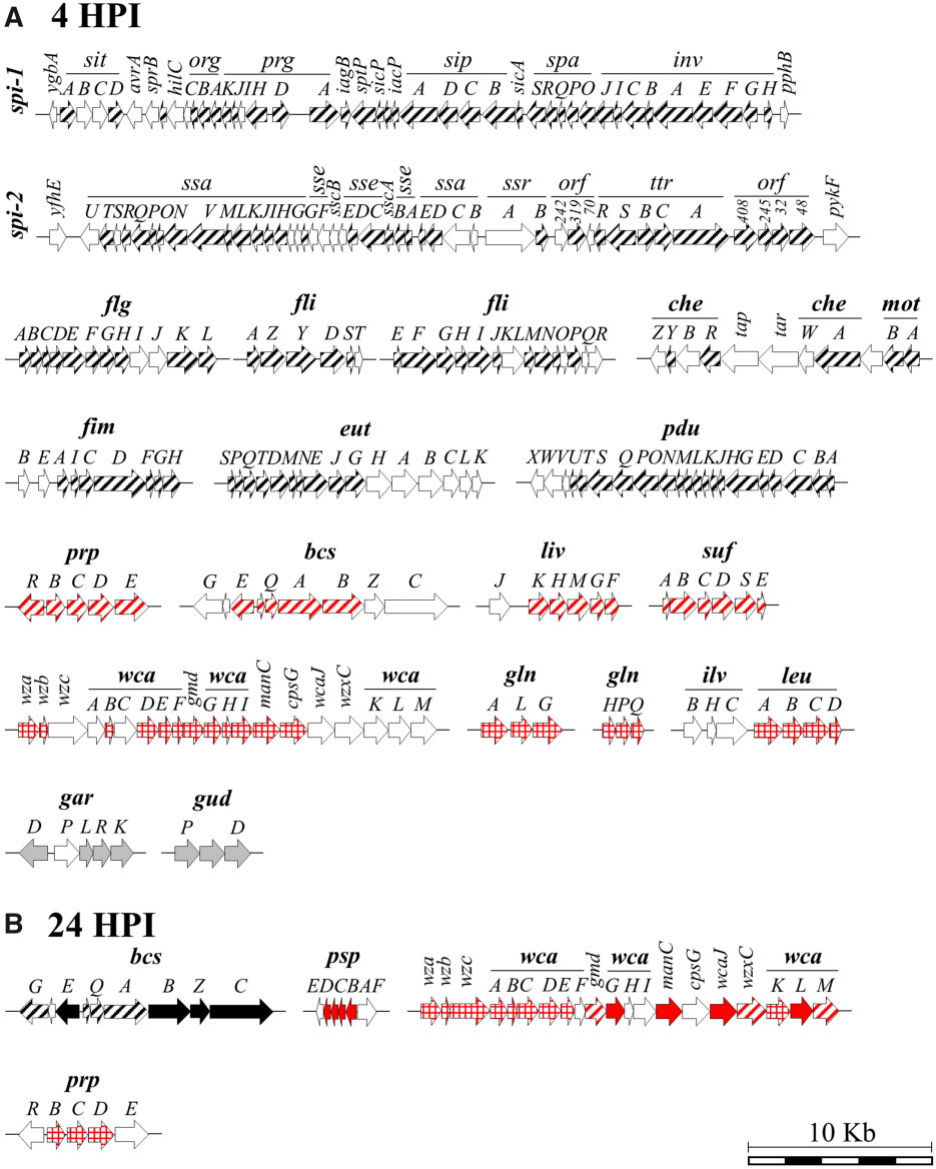
Major operons and gene clusters significantly modulated in *Salmonella enterica* ser. Typhimurium 14028s at 4 h (A) and 24 h (B) post inoculation (HPI). *Salmonella* pathogenicity island-1 (*spi-1*) and -2 (*spi-2*), flagella (*flg and fli*), chemotaxis and motility (*che*-*mot*), fimbria (*fim*), ethanolamine (*eut*), propanediol (*pdu*), propionate (*prp*), bacterial cellulose synthase (*bcs*), leucine, isoleucine, and valine transport system (*liv*), sulfur formation system (*suf*), colanic acid (*wca*), glutamine synthase (*glnALG*), glutamine transporter (*glnHPQ*), leucine biosynthesis (*ilv*-*leu*), D-galactarate (*gar*), D-glucarate (*gud*), and phage shock proteins (*psp*). Genes differentially expressed in lettuce only (squared), Arabidopsis only (striped), and both lettuce and Arabidopsis (filled) are depicted within each operon and gene cluster. Genes colored in red, black, or white indicate upregulation, downregulation, or not differentially expressed, respectively. Genes colored in gray showed opposite expression patterns in each plant species (operons *gar and gud* were induced in lettuce and repressed in Arabidopsis). Schematic representation of operons and gene clusters was created based on the Ensembl Bacteria (https://bacteria.ensembl.org/index.html) genome browser. Description of the operons can be found in Dataset S11.

Both O157:H7 and STm 14028s exhibited an upregulation of the genes of the phage shock protein *psp* operon at 24 HPI in both lettuce and Arabidopsis (Figures 6 and 7). The phage shock protein system is synthesized not only in response to phage infection, but also under multiple stressors (Flores-Kim and Darwin 2016). This seems to be a common bacterial response to the plant environment, as one of the largest group of genes upregulated in *E. coli* K-12 after spray inoculation on lettuce leaves included the *psp* operon (Fink *et al.* 2012).

We also observed multiple gene clusters that were modulated in O157:H7 and/or STm 14028s in a plant-dependent manner (Dataset S11), which are discussed below.

##### Influence of the plant species on the expression of O157:H7 operons

Operons modulated in both plant species. O157:H7 exhibited a strong regulation of nitrogen metabolism during exposure to the leaf apoplast (Figure 5). For example, the four highest upregulated genes in both plant species are part of the seven-gene operon *rutABCDEFG* that encodes a pathway to derive nitrogen from the degradation of pyrimidines (Figure 6; Parales and Ingraham 2010). O157:H7 also stimulated the expression of the *glnALG* operon and the nitrogen regulatory protein P-II 1 (*XF37_18635*; Dataset S11) that is involved in response to nitrogen starvation (Javelle and Merrick 2005), as well as the induction of the *trp* operon that controls tryptophan biosynthesis under changes in the nutritional environment (Figure 6; Yanofsky and Horn 1994). Moreover, O157:H7 upregulated the complete *liv* (leucine/isoleucine/valine transport system) operon (Figure 6; Bhagwat *et al.* 1997). These results reveal a common metabolic adaptation of O157:H7 to the nitrogen environment of the leaf apoplast of lettuce and Arabidopsis.

Sulfur metabolism was enriched in both plant species in O157:H7 (Figure 5), upregulating both *tauABCD and ssuEADCB* operons (Figure 6). The gene clusters *tauABCD and ssuEADCB* are required for the bacterial utilization of taurine and alkanesulfonates as sulfur sources and are expressed only under conditions of sulfate or cysteine starvation (Eichhorn *et al.* 2000). The expression of these gene clusters suggests a potential sulfate or cysteine limitation in Arabidopsis and lettuce leaves for the metabolism of O157:H7.

Operons uniquely modulated in Arabidopsis. A much larger number of O157:H7 operons and metabolic pathways were modulated in the transition to Arabidopsis than lettuce (*i*.*e*., 4 HPI *vs* inoculum; Figure 5; Dataset S11). The lsr (*luxS* regulated) operon was induced in O157:H7 (Figure 6). This operon is responsible of the uptake and processing of the quorum sensing autoinducer-2 (AI-2) molecule that controls genes involved in virulence factors (Pereira *et al.* 2013). Notably, operons involved in bacterial virulence mechanisms were significantly downregulated, including the locus of enterocyte effacement (*lee1-7*) (Gaytán *et al.* 2016), the type three secretion system apparatus and effectors gene cluster (*TTSS-2*; Zhou *et al.* 2014), the chemotaxis and motility (*che-mot*) operon (Ditty *et al.* 1998), the flagellar (*flg and fli*; Liu and Ochman 2007) and fimbrial (*fim*) formation operons, and the CA gene cluster (*wca*; Wu *et al.* 2019) (Figure 6). Interestingly, virulence factors of O157:H7 (*i*.*e*., LEE and TTSS) have shown a positive role in the colonization of spinach after surface inoculation (Saldaña *et al.* 2011), which suggest that the participation of these elements on plant colonization by O157:H7 might be dependent on experimental factors, such as inoculation method.

Significant repression of genes related to energy generation occurred in O157:H7 (Figure 5 and Supplementary Figure S12, A and B). *E. coli* and related enterobacteria can couple the reduction of nitrate and nitrite to ammonium to energy-conserving respiratory electron transport systems, which occurs in many electron-rich environments such as the human gastrointestinal tract (Cole and Richardson 2008). In Arabidopsis, O157:H7 repressed the whole nitrate reductase-cytochrome c maturation (*nap-ccm*) locus (Stewart *et al.* 2002) and the respiratory nitrite reductase gene cluster *nrfABCDEFG* (Wang and Gunsalus 2000; Supplementary Figure S12, A and B). Similarly, after spray inoculation on lettuce leaves, *E. coli* K-12 largely downregulated processes involved in energy metabolism, including nitrate reductase genes (Fink *et al.* 2012).

Genes involved in the utilization of certain carbon sources were significantly modulated only in Arabidopsis (Figure 5). For instance, O157:H7 induced the propionate *prpRBCDE* operon at 4 HPI (Figures 5 and 6), which metabolizes propionate via the 2-methylcitric acid cycle that yields pyruvate and succinate (Horswill and Escalante-Semerena 2001). Propionate is among the most abundant nutrients present in the colon and it is degraded by several members of the gut microbiota (Rivera-Chávez and Bäumler 2015).

Operons uniquely modulated in lettuce. O157:H7 genes with the highest Log_2_ FC were those in the *phn* operon (Figure 6; Dataset S11), which is responsible for organophosphonate utilization (Jochimsen *et al.* 2011). In addition, O157:H7 induced the genes of the high-affinity phosphate transport system *pstSCAB-phoU* operon (Figure 6) and the two-component regulatory mechanism proteins PhoR (*XF37_18915*) and PhoB (*XF37_18920*; Dataset S11), which are activated during phosphate starvation to regulate phosphate homeostasis (Gardner *et al.* 2014). Furthermore, various pathways involved in amino acid metabolism were exclusively significantly enriched by O157:H7 in lettuce (Figure 5). The *ilv* operon, which encodes enzymes forming the isoleucine and valine biosynthetic pathway (Wechsler and Adelberg 1969), and the glutamine ABC transporter (*glnHPQ*) operon were only induced in O157:H7 inoculated into lettuce (Figure 6; Dataset S11). These findings suggest plant specificity in substrate utilization by O157:H7. A similar observation has been reported in other plant systems where genes associated with metabolic responses were differentially modulated during O157:H7 growth in lettuce and spinach cell wall polysaccharides, spinach leaf lysates, and root exudates (Crozier et al. 2016). The study conducted by Kyle *et al.* (2010) also showed a significant modulation of various genes involved in the carbohydrate transport systems in O157:H7 when growing in lettuce leaf lysates, revealing the bacterial availability to use carbohydrates released from injured plant cells.

##### Influence of the plant species on the expression of STm 14028s operons

Operons modulated in both plant species. Certain operons involved in carbon and nitrogen metabolism were commonly modulated in lettuce and Arabidopsis by STm 14028s (Figure 5; Dataset S11). Similar to O157:H7, STm 14028s induced the propionate *prpRBCDE* operon at 4 HPI in Arabidopsis and at 24 HPI in lettuce (Figure 7), which metabolizes propionate via the 2-methylcitric acid cycle that yields pyruvate and succinate (Horswill and Escalante-Semerena 2001). In addition, genes belonging to the operons *gar and gud*, responsible of the degradation and utilization of D-galactarate and D-glucarate as a source of carbon (Monterrubio *et al.* 2000), were repressed by STm 14028s in Arabidopsis while induced in lettuce at 4 HPI (Figure 7). Furthermore, the expression of the two genes encoding glutamate synthase (*MC58_20705* and *MC58_20710*; Dataset S11), an enzyme also involved in the bacterial assimilation of nitrogen (van Heeswijk *et al.* 2013), was repressed in Arabidopsis and induced in lettuce.

Genes within operons involved in biofilm formation were significantly modulated. For instance, STm 14028s induced genes of the CA (*wca*) gene cluster in both plants at 24 HPI, whereas induction of these genes started at 4 HPI in lettuce (Figure 7). In addition, this bacterium repressed genes of the bacterial cellulose synthesis (*bcs*) operon (Römling and Galperi 2015) in both plant species at 24 HPI (Figure 7). The role in attachment and persistence of genes involved in the formation of the extracellular matrix has been studied in multiple plant species (Yaron and Römling 2014). For instance, mutants of *S*. *enterica* ser. Enteritidis with deletions in the *bcsA* or *wcaD* genes exhibited a significant reduction in the attachment to and survival on alfalfa sprouts (Barak *et al.* 2007).

Operons uniquely modulated in Arabidopsis. In Arabidopsis, STm 14028s repressed several genes comprising the *Salmonella* pathogenicity islands 1 and 2 (SPI-1 and SPI-2; Figure 7), which enable the efficient penetration of the intestinal epithelium (Phoebe *et al.* 2001; Figueira and Holden 2012). Similar to O157:H7, we detected repression of multiple STm 14028s genes comprising the flagella (*flg and fli*; Liu and Ochman 2007) and the chemotaxis gene clusters (*che*-*mot*; Ditty *et al.* 1998), together with genes encoding fimbrial (*fim*) proteins (Figure 7). Zarkani *et al.* (2020) also reported that STm 14028s generally repressed the flagellin production during the colonization of tomato plants.

Genes involved in the utilization of certain carbon sources and the transport of amino acids were also significantly modulated only in Arabidopsis (Figure 5). Ethanolamine and 1,2-propanediol can be utilized by *S. enterica* as a sole carbon source by inducing, respectively, the ethanolamine (*eut*) and propanediol (*pdu*) utilization operons (Srikumar and Fuchs 2011). The genes most repressed by STm 14028s included 18 genes of the 23-gene *pdu* operon and various genes of the *eut* operon were repressed (Figure 7). Ethanolamine is an important source of carbon and/or nitrogen for pathogenic bacteria, it is particularly prevalent in the gastrointestinal tract, and it contributes to infection and colonization in the host (Rivera-Chávez and Bäumler 2015; Kaval and Garsin 2018). Furthermore, STm 14028s upregulated most of the genes comprising the *liv* operon only in Arabidopsis (Figure 7).

Similar as in O157:H7, multiple genes involved in energy generation were repressed by STm 14028s (Figure 5 and Supplementary Figure S12, C and D). Energy production involves respiratory chains that mainly consist of dehydrogenases and terminal reductases or oxidases that are linked by quinones (Unden *et al.* 2014). STm 14028s downregulated 12 genes in Arabidopsis belonging to the NADH dehydrogenase A/B gene operon, the entire *atp* operon, which encodes for the bacterial F1F0-ATP synthase and includes the genes ATP synthase subunit IACBδαγβε (Preiss *et al.* 2015), and the whole *frdABCD* operon, which encodes the fumarate reductase enzyme complex (Supplementary Figure S12A). A larger number of genes related to respiration were suppressed in STm 14028s than that in O157:H7 in Arabidopsis (Figure 5 and Supplementary Figure S12).

In Arabidopsis, STm 14028s stimulated the expression of multiple genes involved in response to oxidative stress such as flavin mononucleotide (FMN)-dependent NADH-azoreductase (*MC58_07180*), glutaredoxin (*MC58_10275*), peroxidase (*MC58_16865*), alkyl hydroperoxide reductase (*MC58_11560*), and thioredoxin reductase (*MC58_10035*) (Dataset S11). In addition, the *sufABCDSE* operon that encodes the sulfur formation system stimulated under iron limitation and oxidative stress (Ayala-Castro *et al.* 2008) was induced (Figure 7). It has been suggested that the response of *Salmonella* ser. Newport to oxidative stress induced in tomato plants is crucial for the survival of bacteria in the plant environment (Ferelli *et al.* 2020).

Operons uniquely modulated in lettuce. Similar to O157:H7, STm 14028s induced the expression of the *glnALG* operon, in addition to stimulating the glutamine ABC transporter (*glnHPQ*) operon in lettuce (Figure 7). Glutamine synthetase is key for the bacterial assimilation of nitrogen and the *glnALG* operon, which encodes two nitrogen regulation proteins, NR(I) and NR(II) and a glutamine synthetase (van Heeswijk *et al.* 2013), is induced under limiting nitrogen sources (Ueno-Nishio *et al.* 1983). The two most upregulated genes by STm 14028s in lettuce were the ones encoding nitrogen regulatory protein P-II 1 (*MC58_12240*) and the ammonium transporter (*MC58_12235*; Dataset S11). Furthermore, the operon encoding the genes for the biosynthesis of leucine (*ilv*-*leu*), which is regulated in response to leucine availability (Grandoni *et al.* 1992), was induced only in lettuce (Figure 7; Dataset S11).

Altogether, these results indicate that STm 14028s and O157:H7 exhibit common and unique responses to the apoplast of lettuce and Arabidopsis leaves, which might have a role in the differential capacity of these human pathogenic bacteria to persist inside the leaf. Furthermore, these responses suggest the existence of specific modulation of their metabolism for survival in an environment that is substantially different from their natural niche, the gastrointestinal tract (Hirt 2020).

## Conclusions

I. The transcriptomic analyses enabled the discovery of common and specific modulation of biological processes in Arabidopsis and lettuce upon stimulation by STm 14028s or O157:H7 that are bacterial species- and time-dependent.
II. Plant transcriptomic readjustments after infiltration with STm 14028s or O157:H7 are characterized by an overall downregulation of genes involved in photosynthesis and upregulation of genes associated to responses against biotic stressors. These results agree with the growth–defense tradeoffs commonly observed during plant–pathogen interactions.
III. Human pathogenic bacteria cause more transcriptomic changes in Arabidopsis than in lettuce. This finding exposes the significant effect of plant genotypic variation in the interaction between the leaf and human pathogenic bacteria at the genome-wide level.
IV. At 24 HPI, STm 14028s induces an overall larger modulation of transcription in lettuce and Arabidopsis as compared to that of O157:H7. A stronger stimulation of defense responses by STm 14028s than O157:H7 was observed, which was associated with a lower capability of STm 14028s to persist in the leaf apoplast than O157:H7, revealing a variation in the potential risk of contamination of leaves by different species of human pathogenic bacteria.
V. O157:H7 and STm 14028s modulation of metabolic processes in leaves exhibited specificities and commonalities that are dependent upon plant species and time after inoculation.
VI. Stimulation of changes in gene expression at the whole genome level in STm 14028s and O157:H7 is substantially larger in the first 4 h as compared to the subsequent 20 h after inoculation. This suggests a rapid reaction of the bacteria to the leaf apoplast environment and that most of these initial adjustments are maintained for at least the following 20 h.
VII. Bacterial transcriptomic responses are substantially more extensive in Arabidopsis than in lettuce and modulation of gene expression is larger in STm 14028s than O157:H7. These results agree with the larger counter response induced in Arabidopsis than in lettuce. Moreover, variation in bacterial DEGs might have key roles in the differential ability of STm 14028s and O157:H7 to persist in the leaf apoplast between the two plant species.
VIII. This study generated extensive functional genomics resources that can be further explored to test relevant hypothesis in the future.

## Data availability

Raw sequencing data are available at the National Center for Biotechnology Information Short Read Archive, under the BioProject accession code PRJNA634120. Lists of DEGs and enriched GO terms of the treatments are listed in Dataset S01 and Dataset S02, respectively. Supplemental material available at figshare: https://doi.org/10.25387/g3.16578644.
